# Harness the synergy between targeted therapy and immunotherapy: what have we learned and where are we headed?

**DOI:** 10.18632/oncotarget.21160

**Published:** 2017-09-22

**Authors:** Xiaoyan Liu, Qing Zhou, Yan Xu, Minjiang Chen, Jing Zhao, Mengzhao Wang

**Affiliations:** ^1^ Department of Pulmonary Medicine, Lung Cancer Center, Peking Union Medical College Hospital, Chinese Academy of Medical Sciences, Beijing, People's Republic of China

**Keywords:** immunotherapy, targeted therapy, immune effects, combined therapy, synergistic effects

## Abstract

Since the introduction of imatinib for the treatment of chronic myelogenous leukemia, several oncogenic mutations have been identified in various malignancies that can serve as targets for therapy. More recently, a deeper insight into the mechanism of antitumor immunity and tumor immunoevasion have facilitated the development of novel immunotherapy agents. Certain targeted agents have the ability of inhibiting tumor growth without causing severe lymphocytopenia and amplifying antitumor immune response by increasing tumor antigenicity, enhancing intratumoral T cell infiltration, and altering the tumor immune microenvironment, which provides a rationale for combining targeted therapy with immunotherapy. Targeted therapy can elicit dramatic responses in selected patients by interfering with the tumor-intrinsic driver mutations. But in most cases, resistance will occur over a relatively short period of time. In contrast, immunotherapy can yield durable, albeit generally mild, responses in several tumor types via unleashing host antitumor immunity. Thus, combination approaches might be able to induce a rapid tumor regression and a prolonged duration of response. We examine the available evidence regarding immune effects of targeted therapy, and review preclinical and clinical studies on the combination of targeted therapy and immunotherapy for cancer treatment. Furthermore, we discuss challenges of the combined therapy and highlight the need for continued translational research.

## INTRODUCTION

The last decade has witnessed an unprecedented advance in the medical treatment of cancer thanks to the rapid development of targeted therapy and the recent revival of immunotherapy. Targeted agents can specifically inhibit oncogenic signaling in cancer cells and accomplish striking tumor responses in molecularly defined subsets of patients, although initial regression are commonly followed by the development of progressive diseases [1]. In parallel to these advances in targeting genetic drivers of tumorigenesis, another area of success in the treatment of cancer centers on the exploration of the mechanisms of tumor immunoevasion that has led to the development of several novel agents–most notably the immune checkpoint inhibitors which reverse T cell inhibition with antibodies against the programmed cell death protein-1 (PD-1) or its ligand programmed cell death-ligand 1 (PD-L1) and cytotoxic T-lymphocyte–associated antigen 4 (CTLA-4)–that show durable efficacy in subsets of patients with diverse tumor types and could achieve disease control for extended periods [2]. In spite of these advantages, a deficiency of treatment with immune checkpoint inhibitor is the relatively low response rate in some tumor types because of the lack of definite predictive biomarkers. In addition, immunosuppressive effects of heavy baseline tumor burden may also limit efficacy of immunotherapy [3]. Given the dramatic tumor regression elicited by targeted therapy, as well as its immune potentiating effects [4], it is reasonable to explore the potential synergistic combination of targeted and immune therapy, which will hopefully yield a high and prolonged response. This review examines the immune-based mechanisms of targeted agents, presents available data related to the use of combined targeted- and immunotherapy, and discusses the challenges we face in the clinical application of the combination therapy.

### Immune effects of targeted therapy

**Figure 1:**
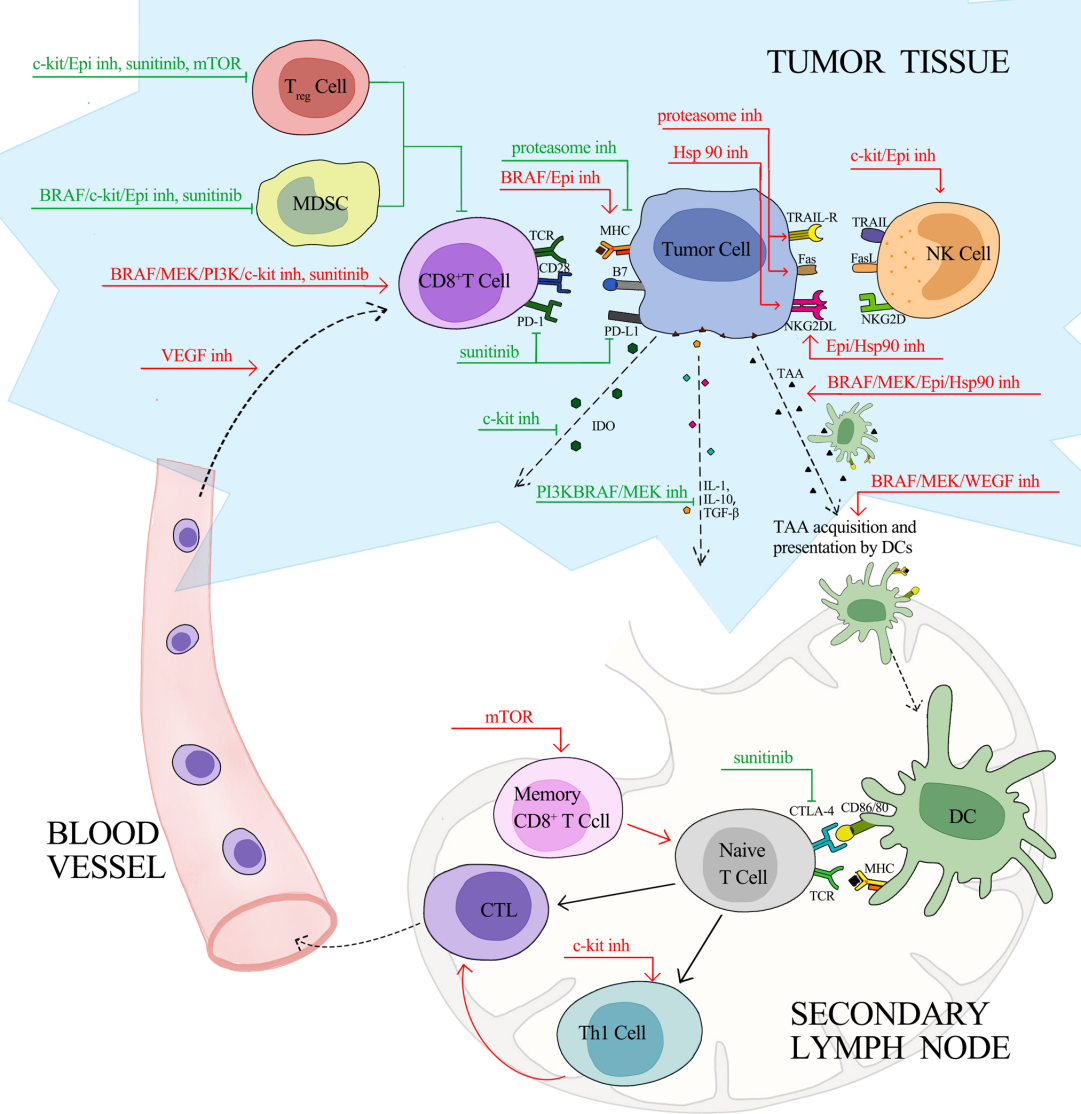
Effects of Targeted Therapy on Anti-Cancer Immunity Red line, stimulatory effects; green line, inhibitory effects; inh, inhibitor; TAA, tumor associated antigen; TRAIL, TNF-related apoptosis-inducing ligand; TRAIL-R, TNF-related apoptosis-inducing ligand receptor; NK, natural killer; NKG2D, natural killer group 2, member D; NKG2DL, natural killer group 2, member D ligands; TCR, T-cell receptor; MHC, major histocompatibility complex; CTLA-4, cytotoxic T-lymphocyte-associated protein 4; PD-1, programmed death 1; PD-L1, programmed death-ligand 1; IDO, indoleamine 2, 3-dioxygenase; IL, Interleukin; TGF, transforming growth factor; DC, dendritic cell; Th1, T helper 1; CTL, cytotoxic lymphocyte; Treg, regulatory T cell; MDSC, myeloid-derived suppressor cell.

Targeted therapy has changed the treatment paradigm of cancer over the past decades. Apart from conventionally proposed mechanisms of inhibiting driver mutation in cancer cells, targeted agents can produce immunoactivating effects via bolstering tumor cells immunogenicity, enhancing immune cells effector function, and relieving tumor-mediated immunosuppression (Table 1). Several key steps are involved in an efficient anti-tumor response. First, tumor associated antigens released from immunogenic cell death are taken up processed by dendritic cells (DCs) in the tumor microenvironment. Then, DCs migrate to tumor-draining lymph nodes where they promote the priming and differentiation of naïve T cells. Subsequently, effector T cells travel from the lymph nodes through blood vessels to the tumor bed, and recognize and eliminate the tumor cell. As illustrated in Figure 1, targeted agents have a broad impact on the complex signals involved in the multistep process. Monoclonal antibodies (mAbs), which attach to and block the extracellular ligand binding domain of targeted kinase, play a direct immunoactivating role by antibody-dependent cellular cytotoxicity (ADCC). Thus, mAbs will not be further discussed here. In the following paragraph, we mainly discuss immune effect induced by small molecule inhibitors targeting different oncogenic signaling pathways.

**Table 1:** Immune-based mechanisms of targeted therapy

Pathway inhibition	Immune Potentiating Effects	Refs
**PI3K/AKT/mTOR pathway**		
PI3K inhibition	Heighten the antitumor properties of TLR ligands Increase accumulation of effector T cells Dampen Treg function Inhibit myeloid cells Inhibit immunosuppressive TAMs	[7] [7] [8] [8] [8]
AKT inhibition	Sensitize tumor cells to immune destruction by disrupting Mcl-1 mediated anti-apoptotic signaling	[10,11]
mTOR inhibition	Generate memory CD8 + T cells Reduce Tregs	[13–16] [13–16]
**MAPK pathway**		
BRAF inhibition	Directly enhance T cell function Increase antigen expression Increase MHC class I expression Restore IL-12 and TNF-α production by DCs Restore CD80, CD83, and CD86 expression on DC Reduce MDSCs Reduce the expression of VEGF Suppress the expression of IL-1 Increase CD8 T cell and NK cells	[31] [33–35] [36] [37] [37] [38] [39] [40] [41]
MEK inhibition	Protect effector CD8 T cells from death caused by chronic T cell receptor stimulation Increase antigen expression Restore IL-12 and TNF-α production by DCs	[32] [34] [37]
**VEGF pathway**		
VEGF/VEGFR inhibition	Increase extravasation of T cell Augment DC maturation and function	[65, 66] [59, 60]
Multikinase inhibition	Decrease the number and function of MDSCs and Tregs Increase cytotoxic lymphocyte infiltration and response Enhance IFN-gamma production Diminish expression of CTLA4, PD1, and PDL-1	[69–71] [69–71] [69–71] [69–71]
**C-kit pathway**		
C-kit inhibition	Facilitate production of Th1 cytokine Prompt NK cell activation Suppress IDO production Inhibit Treg and MDSCs	[84, 85, 90] [86, 87] [89] [88–90]
**Epigenetic pathway**		
Epigenetic inhibition	Increase tumor antigen expression Increase MHC molecules expression Induce the expression of NKG2DL (MICA/B) Reduce Treg cells and MDSCs	[109–111] [109–111] [112, 113] [114, 115]
**Others**		
Proteasome inhibition	Decrease expression of peptide–MHC class I complex (thereby sensitizing tumor to NK cells) Increase expression of FAS and the TRAIL receptor DR5 Induce NOXA-mediated enhancement of mitochondrial SMAC release (thereby increasing sensitivity to T cells)	[130] [131] [132]
HSP90 inhibition	Increase tumor antigen presentation Augment the expression of NKG2DL (MICA/B)	[135, 136] [137, 138]

Abbreviations: TAMs, tumor-associated macrophages; TLR, toll-like receptor; Mcl-1, myeloid cell leukemia-1; Treg, regulatory T cell; MHC, major histocompatibility complex; IL, Interleukin; TNF--α, tumor necrosis factor-α; DCs, dendritic cells; NK, natural killer; MDSCs, myeloid-derived suppressor cells; CTLA-4, cytotoxic T-lymphocyte-associated protein 4; PD-1, programmed death 1; PD-L1, programmed death-ligand 1; Th1, T helper 1; IDO, indoleamine 2, 3-dioxygenase; NKG2DL, natural killer group 2, member D ligands; MICA/B, major histocompatibility complex class I related-A and –B; TRAIL, TNF-related apoptosis-inducing ligand.

### PI3K/AKT/mTOR pathway inhibitors

The phosphatidylinositol 3-kinase (PI3K)/AKT/mechanistic target of rapamycin (mTOR) pathway is an intracellular signaling pathway that plays a crucial role in regulating cell survival and proliferation. Its dysregulation, which is implicated in a wide range of cancers, occurs via abnormal expression of pathway receptors and/or genetic mutations that lead to constitutive activation of key kinases in the pathway which could sever as targets for drug development [5].

PI3Ks are expressed in a broad range of cells, including all subtypes of leukocytes [6]. Therefore, PI3K inhibition could yield immune modulatory effects. Preclinical studies in different cancer models showed that PI3K inhibitor could heighten the antitumor properties of toll-like receptor (TLR) ligands and was associated with increased accumulation of polyfunctional T cells that secreted multiple effector cytokines, including interferon-γ (IFN-γ), interleukin-17 (IL-17), and IL-2 [7]. In addition, PI3K inhibition was shown to dampen Treg function, inhibit suppressive myeloid cells, and dampen immunosuppressive tumor-associated macrophages (TAMs) [8]. The activation of Akt was shown to increase programmed death ligand-1 (PD-L1) expression and lead to immunoresistance which could be reversed by inhibition of PI3K and mTOR [9]. In addition, AKT inhibition sensitized tumor cells to immune destruction by disrupting Mcl-1 mediated anti-apoptotic signaling [10, 11]. In an immune-resistant human papillomavirus type 16 (HPV-16) E7-expressing tumor cell line, AKT was shown to be over-activated. Consistently, retroviral transfer of a constitutively active form of AKT induced resistance against E7-specific CD8+ T-cell mediated apoptosis, which was associated with the upregulation of anti-apoptotic molecules. Besides, intratumoral injection of an AKT inhibitor enhanced the efficacy of immunotherapy [10]. Other studies also demonstrated that activation of AKT, by preventing apoptosis, induced resistance against the cytotoxicity of both antigen-specific cytotoxic T-cell lymphocytes (CTLs) *in vitro* and adoptively transferred cellular immune effectors *in vivo* [11].

The role of mTOR in the regulation of immune cell function has been well characterized. MTOR inhibitors are widely used to suppress a rejection response by the immune system in organ transplantation [12]. Paradoxically, mTOR inhibitor was recently shown to potentiate immune response by generating memory CD8+ T-cells in a dose- and duration-dependent manner [13–16]. Li et al. [17] reported that a short course of high-dose, instead of low-dose rapamycin, generated memory CD8+ T-cell responses, and afforded more durable protection against tumor compared with persistent administration of either low or high dose. Besides, mTOR inhibitors may have a dual impact on FOXP3+ regulatory T (Treg) cells. Current evidence showed that transient mTOR inhibition with rapamycin promoted T cell receptor (TCR)-induced Treg cells proliferation before TCR stimulation [18], whereas prolonged inhibition led to significant Treg cells depletion [19]. Based on these findings, several investigations explored the potential synergy between mTOR inhibitors and cancer immunotherapy. In a murine model of metastatic renal cell carcinoma (mRCC), a combination of the adenosine triphosphate (ATP) -competitive mTOR kinase inhibitory agent AZD8055 and the alphaCD40 agonistic antibody yielded synergistic antitumor responses. The addition of AZD8055 increased the proliferation and activation of CD8 T-cells and natural killer cells, as well as matured macrophages and dendritic cells [20]. Another study on a murine model of RCC (RENCA) and melanoma (B16) showed that combined treatment with heat shock protein (HSP)-based cancer vaccines and temsirolimus augmented interferon-γ production and cytotoxic T-cell responses and enhanced generation of CD8 memory cells [21]. These preclinical results provide a strong rationale for further exploiting mechanisms by which PI3K/AKT/mTOR pathway inhibitors modulate antitumor immune response, thereby better guiding the clinical design, particularly in cancers harboring PI3K mutations, such as glioblastomas, breast, colon and endometrial cancers [22].

### MAPK pathway inhibitors

The mitogen-activated protein kinase (MAPK) pathway includes the signaling molecules Ras, Raf, MEK, and ERK, and functions in the regulation of gene expression, cellular proliferation, and differentiation, and survival [23]. Abnormal MAPK signaling contributes to uncontrolled cell growth and resistance to apoptosis and is implicated in a wide range of cancer [24]. Besides, MAPK pathway also participates in the regulation of T cell expansion and differentiation, as well as T cell functions, including cytokine secretion and chemotaxis [25]. Two signals are the sine qua non for T cell activation: binding of the TCR to the antigen-major histocompatibility complex (MHC) and engagement of co-stimulatory molecules, which in turn, will activate downstream signal transduction cascades including MAPK pathway [25, 26]. Thus, MAPK pathway activation enhances T-cell proliferation and function. Conversely, its inhibition dampens T-cell response.

Besides acting on tumor cells carrying the RAF mutation, RAF inhibitors can also stimulate T cell-mediated immunity against tumors. Accumulating evidence suggests that RAF inhibitors have opposing effects on BRAF-mutant and BRAF wild-type cells. While RAF inhibitors block ERK signaling in BRAF-mutant cells, they paradoxically enhance ERK signaling in BRAF wild-type cells in preclinical studies [27, 28]. It is further validated by the clinical observation of treatment-induced squamous cell cancer and keratoakanthomas in patients receiving RAF inhibitors [29, 30]. In a BRAF V600E-driven murine model of melanoma, combined treatment with vemurafenib and TCR engineered adoptive cell therapy (ACT) resulted in superior antitumor responses compared with either therapy alone [31]. And vemurafenib increased intratumoral cytokine secretion and *in vivo* cytotoxic activity of adoptively transferred cells via MAPK activation [31]. Similarly, MEK inhibitors, a recent study in immune-competent mice found that MEK inhibitors protected effector CD8 T-cells from death caused by chronic T cell receptor stimulation while sparing cytotoxic activity [32].

Several studies have demonstrated that inhibition of MAPK pathway by BRAF and MEK inhibitors leads to increased expression of melanocyte differentiation antigens in both melanoma cell lines and clinical tumor samples from melanoma patients [33–35]. Further, loss of tumor antigen expression is observed when resistance to BRAF inhibition develops [35]. Besides, it has also been reported that BRAF inhibition induced upregulation of MHC class I expression in tumor cells and facilitated antigen presentation and recognition [36]. Besides, blockade of the MAPK pathway may enhance dendritic cell function. *In vitro* showed that MEK and BRAF inhibitors could restore the decreased production of IL-12 and tumor necrosis factor-α (TNF-α) by DCs co-cultured with BRAFV600E mutant cells [37]. Moreover, the CD80, CD83, and CD86 expression on DC was reduced upon co-culture and can be partially restored with BRAF inhibition.

Additional mechanisms by which MAPK pathway inhibitors bolster antitumor immunity involve improving the tumor microenvironment by reducing the suppressive immune cells and cytokines. In the blood sample of melanoma patients, vemurafenib, a BRAF inhibitor, decreased the frequency of monocytic myeloid-derived suppressor cells (MDSCs). And it inhibited MDSCs generation in an *in vitro* model of the melanoma microenvironment [38]. Furthermore, BRAF inhibitor can increase intratumoral infiltration and antitumor activity of TCR-engineered ACT by inhibiting tumor cell production of the vascular endothelial growth factor (VEGF) in xenograft mouse model [39]. In consistent, decreased VEGF expression was also observed in tumor biopsies of patients receiving BRAF inhibitor treatment [39]. BRAF inhibitors also reduce IL-1 expression in cell lines and tumor biopsies, which in turn may, as suggested by Khalili et al. [40], theoretically be able to relieve T cell suppression caused by IL-1 mediated upregulation of PD-1 ligands and COX-2 expression on tumor-associated fibroblasts (TAFs). Another study in BRAF V600E mouse melanoma transplants and in de novo melanomas demonstrated that BRAF inhibitor downregulated tumor expression of chemokine (C-C motif) ligand 2 (CCL2), and resulted in a robust increase in CD8+ T/FoxP3+CD4+ T cell ratio and natural killer (NK) cells [41].

Multiple studies have reported increased intratumoral T cells infiltration in BRAF mutant melanoma tumors treated with MAPK pathway inhibitors [35, 39, 42]. This may be due to the direct facilitation of T cell trafficking, or secondary to increases in tumor cell antigenicity and immunogenicity. As the aforementioned transient induction of melanoma tumor antigens by BRAF and MEK inhibitors, increase in intratumoral T cells resulted from MAPK pathway inhibition was also lost at the time of progression on therapy [35].

BRAF inhibitors were reported to be associated with an increase in immunosuppressive T cell exhaustion markers TIM-3 and PD1 and its ligand PD-L1 [35], suggesting that combining MAPK targeted therapy with PD1/PD-L1 inhibitors may improve responses. Subsequent studies have provided supporting evidence for the potential synergistic effect of this combination therapy [43, 44]. However, it is still unclear whether the enhanced expression of exhaustion markers resulted from the increase in T cell infiltration and cytotoxic activity or a direct stimulatory effect of MAPK inhibition on the expression of exhaustion markers. Following findings may shed light on this question. Elevated PD-L1 expression was observed in BRAF inhibitor-resistant melanoma cell lines and tumor samples [42, 45, 46]. Furthermore, induction of PD-L1 expression was shown to be limited to cell lines whose resistance to BRAF inhibitor did not depend on reactivation of the MAPK pathway [47]. Together, these studies implied that increased PD-L1 expression might be secondary to enhanced T cell infiltration facilitated by

In summary, BRAF inhibitors contribute to an intensified antitumor response via several different mechanisms: first, direct enhancing of T cell antitumor activity by a paradoxical activation of the MAPK pathway; second, promoting the tumor antigen expression, recognition, and presentation; third, altering the tumor microenvironment by reducing the suppressive immune cells and cytokines.

### VEGF pathway inhibitors

The VEGF family in mammals is composed of five members: VEGF-A, VEGF-B, VEGF-C, VEGF-D and placenta growth factor (PGF), and binds to different receptors, including vascular endothelial growth factor receptor (VEGFR) and Neuropilin-1 (NRP1) [48]. The role of VEGF signaling pathway in tumorigenesis and progression is multifaceted. First, it is implicated in pathological angiogenesis, lymphangiogenesis and vascular permeability [49]. Second, it promotes tumor growth and inhibits apoptosis in an autocrine or paracrine manner [50]. Moreover, it facilitates epithelial–mesenchymal transition of tumor cells [51] and regulates the function of cancer stem cells, independent of angiogenesis [52–54]. Besides, VEGF signaling affects the function of immune cells and fibroblasts that are present in the tumor microenvironment [55]. Drugs that interfere with the VEGF signaling pathway include two types, one targeting VEGF, such as bevacizumab, an anti-VEGF-A antibody and aflibercept, a recombinant fusion protein containing the VEGF-A/B-binding domains of VEGFR1/2; the other targeting VEGFR, including small molecule multikinase inhibitors like sorafenib and sunitinib, as well as anti-VEGFR2 antibody ramucirumab.

The immune modulating effects of VEGF come down to two aspects. On one side, VEGF-A reduces expression of endothelial cell adhesion molecules, which prompts an abnormal tumor vasculature and inhibits the infiltration of T cells and other immune cells [56–58]. On the other side, VEGF acts on receptors expressed on immune cells, thereby directly modulating the immune response. Preclinical studies demonstrated that VEGF-A suppressed dendritic cell differentiation and activity [59–61], promotes the expansion of MDSCs [59], upregulates the checkpoint molecules expression on CD8 T-cells [62], and modulates the proliferation of regulatory T cells [63, 64].

In accordance with the immune biology of VEGF, VEGF signaling blockade treatment shows multifaceted immune stimulatory effects. Studies in the murine model showed that modulation or normalization of tumor vasculature by anti-angiogenic therapy increased extravasation of T cell into tumors [65, 66], and exhibited a synergistic effect with anti-PD1 therapy [67]. Besides, the inhibitory effect of VEGF on DC maturation and function can be reversed by bevacizumab and sorafenib [59, 60]. Bevacizumab administration to patients with lung, breast, and colorectal carcinoma was associated with enhancement of DC maturation and antigen presentation [68]. In contrast, broader immune modulating effects were demonstrated in anti-angiogenic treatment with multikinase inhibitors. Preclinical and clinical studies have shown that sunitinib or sorafenib could decrease the number and function of MDSCs and Treg cells, enhance cytotoxic lymphocyte infiltration and response, increase IFN-gamma production, and diminish expression of CTLA4, PD1, and PDL-1 [69–71]. However, discordant results were reported. Liu et al. described an enhanced infiltration of Treg cells and an up-regulated expression of PD-L1 [72], while Guislain et al reported an augmented expression of PD-1 and an unvaried Treg cells infiltration [73]. Apart from inhibiting VEGFR, the multikinase inhibitor sunitinib also stimulates T cell response by interfering with signal transducer and activator of transcription 3 (STAT3) activation [70, 74].

Clinical trials combining VEGF-A or VEGFR inhibitors with immunotherapies showed enhanced antitumor activity. In a study of advanced metastatic melanoma treated with bevacizumab and ipilimumab combination therapy versus ipilimumab monotherapy, combination therapy increased expression of E-selectin on vessel endothelium, as well as intratumoral immune cell infiltration that was associated with clinical responses [75]. In patients with mRCC treated with atezolizumab (anti-PD-L1) in combination with bevacizumab, intratumoral T cells were increased by bevacizumab treatment alone, and were further increased upon combination with MPDL3280A [76]. Nonetheless, it should be noted that seemingly related small molecule inhibitors might not have identical activities; sorafenib, a multi-kinase inhibitor that inhibits angiogenesis, was shown to be immunosuppressive *in vitro* [77, 78].

### C-kit inhibitors

Imatinib, a selective inhibitor of BCR-Abl and c-kit receptor tyrosine kinase, is a clear example of the success of targeted therapy for chronic myelogenous leukemia (CML) and gastrointestinal stromal tumors (GIST). Besides directly curbing tumor growth, imatinib also exerts both suppressive and stimulatory effects on host immune system.

The imatinib-induced immunosuppression is mediated by the following mechanisms. First, imatinib can impair the generation of dendritic cells (DCs) via reduced phosphorylation of AKT/ PKB Protein kinase B and nuclear accumulation of NFκB, thereby resulting in less efficient priming of CTLs [79]. Clinical observation of incomplete recovery of circulating DC numbers in CML patients treated with imatinib further provided supporting evidence for imatinib-induced dendritopoiesis [80]. Second, imatinib facilitates the conversion of the TAMs from anti-tumor M1 phenotype to pro-tumor M2 phenotype. In addition, imatinib restrains TCR induced T cell proliferation and activation [81, 82], as well as hampers cytokine synthesis by activated CD4 T cells [83].

Despite the above-mentioned immune inhibitory effects, the overall effect of imatinib on host anti-tumor immune response is stimulatory. The immune potentiating activities are mediated by hindering the activity of protein tyrosine kinases (PTKs) expressed on distinct cell types in host immune system. First, by acting on KIT in DCs, imatinib skews immune responses toward the production of T helper 1 (Th1) cytokines which will potentially contribute to tumor elimination [84, 85]. Besides, *in vitro* and *in vivo* studies of GIST showed that imatinib, by blocking KIT signaling in DCs, prompted DC-mediated NK cell activation, and stimulated the production of IFNα from NK cells [86]. Similar findings have been observed in clinical studies that imatinib treatment enhanced IFNγ secretion in GIST patients [86, 87]. In addition to the activation of favorable cross-talk between DC and NK cells, imatinib also activates CD8+ T-cells, induces Treg cells apoptosis, and reduces the expression of the immune suppressive enzyme indoleamine 2, 3-dioxygenase (IDO) through inhibition of oncogenic KIT signaling [88]. Imatinib was shown to have direct inhibitory effects on Treg cells by downregulating expression of the Treg cells master transcription factor FoxP3 and decreasing their number and suppressive capacity [89]. It also reduced MDSCs and restored effector lymphocyte responses [88]. Dasatinib, another KIT tyrosine kinase inhibitor, also could reduce the suppressive functions of MDSC and restore a Th1 response [90], and its antitumor activity can be strongly potentiated by immune stimulation with agonist anti-OX40 antibody therapy [91].

On the basis of the aforementioned preclinical findings, multiple investigations have studied the combined use of c-Kit inhibitor and various immunotherapeutic approaches. In several phase I and phase II trials, concomitant treatment with imatinib and vaccines against BCR–ABL1 epitopes showed an enhanced anti-tumor activity in patients with CML [92, 93]. Other studies explored the combination of imatinib and IFNα or IL-2 in the treatment of CML. Although several studies showed that the addition of IFNα to imatinib led to higher rates of molecular response in CML patients [94, 95], others yielded conflicting results [96, 97]. The difference among these studies might be ascribed to the high discontinuation rates resulted from IFNα related adverse events [97–99]. The combination of imatinib and pegylated IFNα2b has also been investigated in patients with GIST, and interim analysis have shown promising results [100]. As to the combined use of imatinib and immune checkpoint inhibitor, it showed synergistic anticancer effects [88].

### Epigenetic therapies and immunotherapy

Epigenetic regulation refers to the functional change in the genome without altering underlying nucleotide sequence, which is controlled through modifications on DNA and histone that includes methylation, acetylation, phosphorylation, ubiquitination, sumoylation, proline isomerization and ADP ribosylation [101, 102]. Multiple enzymes participate in the process, including DNA methyltransferases (DNMTs), histone deacetylases (HDACs), histone methyltransferases (HMTs), histone demethylases, histone acetyltransferases, ubiquitin ligases and deubiquitinases [102]. Epigenetic downregulation of tumor suppressor genes and upregulation of oncogenes are involved in cancer development, progression and drug resistance [103, 104]. Recent studies have revealed a dynamic interplay between epigenetic modulation and host antitumor immunity, laying the groundwork for the combination of epigenetic therapies and immunotherapy.

Epigenetic regulation of cancer immunity, which concerns both cancer cells and immune cells, includes downregulation of genes expression involved in recognition and eliminations of malignant cells by immune system, such as cytokine gene, MHC genes, and costimulatory genes [105, 106], as well as upregulation of genes involved in immunosuppressive pathways [107, 108]. Ergo, epigenetic therapies have the potential to render malignant cells more sensitive to immunosurveillance, as well as prime the host immune systems to respond to the immunotherapy.

A growing body of evidence suggests that epigenetic agents can heighten tumor immunogenicity by up-regulating the expression of tumor antigens, MHC molecules, and other molecules involved in antigen processing and presentation [109–111], sensitize tumor cells to NK group 2 member D (NKG2D)-mediated cytotoxicity of NK and T cells by inducing expression of MHC class I–related chain A and B (MICA/B) [112, 113], and reduce Treg cells and MDSCs [114, 115]. Correspondingly, HDAC inhibitors can boost the antitumor activity of immunotherapies in both lung cancer cell line [110] and murine model of various solid tumors [116–119]. However, it is noteworthy that epigenetic agents also exert immunosuppressive effects by augmenting Treg cell function, altering the Th1 and Th2 balance, and inhibiting proliferation and viability of lymphocytes [116, 120–122].

As to the effects on the expression of immune checkpoint molecules, it varies among different epigenetic agents. Preclinical studies have demonstrated that class I HDAC inhibitors, which target deacetylases mainly expressed in the nucleus, lead to upregulation of PD-L1 and PD-L2 in human and murine melanoma cell lines [123]. In contrast, the selective HDAC6 inhibitor rocilinostat (which belongs to class II HDAC inhibitors) or HDAC6-specific silencing is shown to cause the downregulation of PD-L1 in CLL patients [123]. *In vitro* and *in vivo* studies have shown that treatment with DNMT inhibitor decitabine gave rise to a dose-dependent upregulation of PD-1, PD-L1, PD-L2 and CTLA-4 expression [124]. Moreover, elevated expression of immune checkpoint genes was observed in patients resistant to DNMT inhibitor, suggesting its potential role in the development of resistance to demethylating agent [124]. A similar result was found that azacytidine (5-aza)-induced demethylation of PD-1 promoter was associated with heightened PD-1 expression and a significantly worse overall response rate [125]. Another study analyzing gene signatures in lung cancer samples has shown that 5-aza up-regulates genes involved in both tumor immune elimination and evasion. In particular, 5-aza up-regulates PD-L1 transcripts and protein expression, suggesting a superior combinatorial effect of the combined use of DNMT inhibitor and PD-L1 inhibitor [126].

Compatible with the aforementioned immune biology of epigenetic agents, combinatorial therapies of epigenetic agents and immunotherapy have shown promising results in preclinical studies. Combinations of CTLA-4 blocking antibody 9H10 with either 5-aza-2’-deoxycytidine (5-aza-CdR) [127] or the DNMT inhibitor guadecitabine [128] was shown to exert robust antitumor activity in syngeneic mouse models of mammary carcinoma TS/A and mesothelioma AB1. In another study on murine models of mammary and colorectal carcinoma, the addition of epigenetic-modulating drugs (5-aza and entinostat) to checkpoint inhibitors (anti-PD-1 and anti-CTLA-4 antibodies) remarkably improved treatment outcomes, curing more than 80% of the tumor-bearing mice. And function studies revealed that the improved antitumor activity might be partially due to the reduction of MDSCs [114].

### Other targeted agents

Bortezomib is the first therapeutic proteasome inhibitor used in multiple myeloma [129]. While multiple mechanisms are likely to be involved, proteasome inhibitors are viewed as functioning mainly by inhibiting the degradation of proteins critical to growth inhibition and apoptosis, thereby allowing for activation of programmed cell death in malignant cells. Aside from the pro-apoptotic effect on tumor cells, bortezomib can sensitize tumor cells to NK cells by decreasing expression of peptide–MHC class I complexes [130] and increasing tumor cell surface expression of FAS and the TNF-related apoptosis-inducing ligand (TRAIL) receptor DR5 [131]. Furthermore, bortezomib may render tumor cells more susceptible to T cell-mediated cytolysis by inducing mitochondrial accumulation of NOXA and in turn potentiating the release of mitochondrial second mitochondria-derived activator of caspase (SMAC) in response to caspase-8 and granzyme B [132]. Accordingly, enhanced specific CTL response was described when combinatorial treatment with bortezomib and vaccine was administered [133].

Heat shock protein 90 (HSP90) is a major chaperone involved in maintaining correct folding of multiple client proteins, including several oncoproteins that regulate cell growth. HSP90 inhibitor disrupts the association between HSP90 and its client proteins, resulting in the degradation of client proteins [134]. As to the immunomodulatory effects, HSP90 inhibitor induces degradation of client proteins, which may be digested to short peptides and then be presented with MHC class I on cancer cells, thereby increasing tumor sensitivity to immune elimination [135, 136]. In addition, HSP90 inhibitors also augment the expression of natural killer group 2D (NKG2D) ligands, facilitating tumor recognition and elimination by NK cells [137, 138].

### Considerations for the clinical development of combined therapy

Combination therapy may integrate the benefits of the high frequency of rapid tumor regressions achievable with targeted therapies with the durable responses induced by immunotherapy. However, some key considerations for such combinations include identifying predictive biomarkers, optimizing dosing regimen and schedule, and minimizing treatment-related toxicities.

First, the top of any treatment algorithms will be the selection of patients for initial therapy. Though it is well known that patients harboring specific genetic alterations respond to corresponding targeted agents, the predictive biomarkers for immunotherapy remain elusive. Moreover, as the effect on host anticancer immunity induced by targeted agents is still less characterized, combining or sequential use of targeted and immune therapy will further complicate the identification of biomarkers for determining patients who will benefit.

Then, optimization of the dosing regimen and schedule is needed to potentiate benefits. Would combining bring a greater survival benefit compared with sequencing of targeted and immune therapy? If sequencing administration is better, then what is the optimal sequence? Will immunotherapy before targeted agents be more beneficial or vice versa? Within the limited data concerning melanoma, ipilimumab given prior to a BRAF inhibitor appears to be more effective in BRAF mutation–positive melanoma. In a retrospective analysis, longer OS was observed when ipilimumab was given prior to a BRAF inhibitor (BRAFi) compared with BRAF inhibitor followed by ipilimumab, or with either agent alone [139]. However, there is a caveat: different criteria were used to select patients for initial ipilimumab or BRAFi therapy. Patients with a poorer prognosis, e.g., rapid disease progression or brain metastases, are more likely to receive upfront BRAFi treatment. The author suggested that the lower efficacy of ipilimumab after BRAFi might be due to the reduction of the tumor melanocytic antigen expression and T-cell infiltration at progression. Another explanation for the worse prognosis of patients receiving initial BRAFi followed by ipilimumab might be that the group of patients, who generally have several unfavorable prognostic features prior to therapy, experience aggressive tumor growth and short survival after progressing on BRAFi therapy and therefore are hardly able to benefit from ipilimumab, which typically requires weeks or months to show response. Notwithstanding these limitations, the data suggested that first-line ipilimumab might be a preferred option for BRAF-mutant metastatic melanoma patients with low risk of rapid progressing disease. However, this is not the case for PD1 inhibitors. PD1 blockade therapy was shown to be associated with high response rates and a rapid tumor regression. Moreover, BRAFi treatment increases T-cell infiltration into the tumor and may upregulate PD-L1 expression, which may result in a tumor microenvironment more predisposed to respond to subsequent anti-PD-1 therapy following initial BRAFi [140, 141]. And recent studies lend support to this hypothesis. In an open-label, randomized phase III study, patients who progressed after both ipilimumab and a BRAFi treatment still had a higher response rate with nivolumab compared with chemotherapy [142]. Similar results were reported in a randomized phase II trial of pembrolizumab [143]. Hence, awareness should be raised that immunotherapeutic agents acting on different immune signaling pathway may have distinct biological and clinical implications when it comes to sequential using with targeted therapy.

Most of the data regarding the sequence of the use of immunotherapy and targeted agents hitherto is derived from studies in melanoma. However, the scenario could vary wildly in other cancer types. For instance, current evidence showed that ipilimumab is effective in both BRAF mutation–positive or wild patients. In contrast, non-small-cell lung carcinoma (NSCLC) harboring EGFR mutations or ALK rearrangements, as found in a recent study, are associated with low rates of concurrent PD-L1 expression and CD8+ T-cells pre- and post-targeted treatment, as well as low response rates to PD-1/PD-L1 inhibitors, which argues against both combinational and sequential use of targeted agents and immune checkpoint inhibitors. In spite of discrepancies among various cancer types, it is anticipated that learnings obtained from melanoma will somehow apply to other tumor types as well.

Last but not least, avoiding toxicity associated with combinatorial therapy is crucial. A phase I trial evaluating concurrent vemurafenib and ipilimumab treatment in patients with BRAF V600 mutation–positive melanoma demonstrated dose-limiting hepatotoxicity and was terminated [144]. And it remains to be further investigated that if the hepatotoxicity resulted from an autoimmune damage caused by the combination of vemurafenib and ipilimumab-induced hyper-immunity, or a direct liver injury inflicted by the combing use of vemurafenib and ipilimumab. The result of this study highlights the importance of rigorous clinical trial aimed at evaluation and optimization of combined use of targeted and immune therapy in order to optimize the overall risk-benefit ratio.

## CONCLUSIONS

It is increasingly clear that targeted agents, which are initially developed to inhibit tumor-intrinsic drivers of growth, have underappreciated but highly relevant effects on antitumor immunity. The aim of combined approaches is to bring the durable clinical benefit of immunotherapy along with the high response rates and rapid remission elicited by targeted therapy. To this end, future research should continue focusing on investigating the complex interplay between targeted agents and immunotherapy and optimizing parameters such as administration timing, dosage, and sequence that may maximize therapeutic index.
